# Association of dietary copper intake with Alzheimer’s disease, brain structure and cognitive function

**DOI:** 10.1016/j.jnha.2026.100868

**Published:** 2026-05-07

**Authors:** Xintong Wu, Yan Chen, Hao Liu, Zhaochen Sun, Ping Sun, Yuping Liu, Ping Shuai, Han Yang, Liangliang Yang, Zhengwei Wan

**Affiliations:** a School of Medicine, University of Electronic Science and Technology of China, Chengdu, China; b Department of Health Management Center and Institute of Health Management, Sichuan Provincial People's Hospital, School of Medicine, University of Electronic Science and Technology of China, Chengdu, China; c School of Public Health, Southwest Medical University, Luzhou, China; d Division of Internal Medicine, Institute of Integrated Traditional Chinese and Western Medicine, West China Hospital, Sichuan University, Chengdu, China

**Keywords:** Copper intake, Alzheimer’s disease, Cognition fiction, Brain health, U-shaped association

## Abstract

**Objectives:**

This study aimed to investigate the relationships between dietary copper intake and the risk of Alzheimer’s disease (AD), cognitive function, and brain structural measures.

**Design:**

A combined prospective cohort and cross-sectional analysis.

**Setting:**

The UK Biobank.

**Participants:**

This analysis included 126,660 participants (56,053 males and 70,607 females) from the UK Biobank who were free of baseline dementia and had complete dietary data. The mean age at baseline was 56.14 ± 7.83 years, and the median dietary copper intake was 1.33 mg/day (IQR: 1.10–1.61).

**Measurements:**

Dietary copper intake was assessed via 24-h dietary recalls. AD diagnosis was obtained from health records. Cognitive function and brain structural measures were derived from neuropsychological assessments and MRI scans, respectively. Cox proportional hazards models with restricted cubic splines were used to examine associations.

**Results:**

Over a median follow-up of 13.34 years, 619 participants developed AD. A nonlinear U-shaped association was observed between copper intake and AD risk (P for nonlinearity <0.001), with the lowest risk at an intake of 1.57 mg/day. Compared to the reference intake (1–2 mg/day), both lower and higher intakes were associated with increased AD risk, with the highest risk at intakes ≥3 mg/day (HR = 2.73, 95% CI: 1.53–4.87). This pattern remained consistent across subgroups stratified by genetic risk. Copper intake also showed significant nonlinear associations with cognitive function and brain structure, with optimal ranges consistently observed between approximately 1.3 and 2.0 mg/day.

**Conclusion:**

Dietary copper intake exhibits a U-shaped association with AD risk, and maintaining intake within an appropriate range (approximately 1.3–2.0 mg/day) may help reduce AD risk and benefit cognitive function and brain health.

## Graphical abstract

**Figure:**
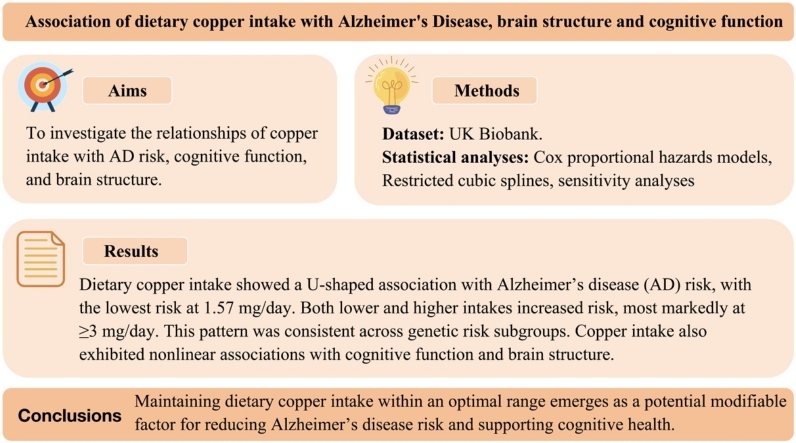

## 1 Introduction

Alzheimer's disease is the most common cause of dementia, accounting for 50%–70% of all cases. Its hallmark symptoms include memory loss, which leads to a decline in cognitive function and significantly impairs daily living activities. As the global population ages, Alzheimer's disease is rapidly becoming one of the most costly, deadly, and burdensome diseases of the 21st century [1]. According to estimates from the International Alzheimer's Association in 2018, approximately 50 million people worldwide are living with dementia, and this number is expected to double by 2050 [2]. Due to the lack of effective treatment for Alzheimer's disease (AD) at present [3], exploring its risk factors and implementing preventive measures has become a key research focus. Recent estimates suggest that up to 40% of dementia cases may be preventable or delayed through the modification of lifestyle and environmental risk factors [4].

Copper is an essential trace element that plays a critical role in maintaining normal brain function, including neurotransmitter synthesis, synaptic transmission, and neuronal metabolism. Dietary copper is obtained primarily from food sources and nutritional supplements. However, current evidence on the relationship between copper intake and cognitive outcomes remains limited and inconsistent. One earlier study reported that higher copper intake, particularly when combined with a diet high in saturated fats, was associated with an increased risk of dementia and cognitive impairment [5]. In contrast, a more recent study found the opposite, suggesting that higher dietary copper intake from food sources may be linked to a lower risk of developing dementia [6]. These conflicting findings indicate that the relationship between copper intake and AD risk may be complex and potentially non-linear. From a biological perspective, dysregulation of copper homeostasis has been implicated in AD pathogenesis. Copper influences various aspects of brain function, including neurotransmitter synthesis, neuropeptide activation, and efficient synaptic transmission in the central nervous system [7]. Dysregulation of copper metabolism is considered a significant contributor to the pathogenesis of AD. Both copper deficiency and excess can impair cellular functions, with copper accumulation in the brain linked to the impaired clearance of Aβ and enhanced tau phosphorylation and aggregation [8]. Together, existing epidemiological and mechanistic evidence supports the hypothesis that deviations from optimal copper intake, may contribute to AD development.

Therefore, the present study aimed to investigate the dose–response association between dietary copper intake and the risk of incident AD in a large prospective cohort of middle-aged and older adults from the United Kingdom. In addition, we examined the associations between dietary copper intake and cognitive performance as well as brain structural measures using neuroimaging data. By integrating epidemiological, cognitive, and neuroimaging evidence, this study seeks to clarify the role of dietary copper intake in AD risk and to inform potential nutritional strategies for AD prevention.

## 2 Material and methods

### 2.1 Study population

The UK Biobank (UKB) is a large population-based prospective cohort study that recruited over 500,000 participants aged 40–69 between 2006 and 2010 across the United Kingdom. The study collected extensive sociodemographic, lifestyle, health, and genetic data through touchscreen questionnaires, interviews, physical measurements, and biological samples, including blood for genotyping. All participants provided written informed consent, and the study protocol was approved by the Northwest Multi-Centre Research Ethics Committee. Follow-up data are obtained through linkage to national electronic health records.

**Fig. 1:**
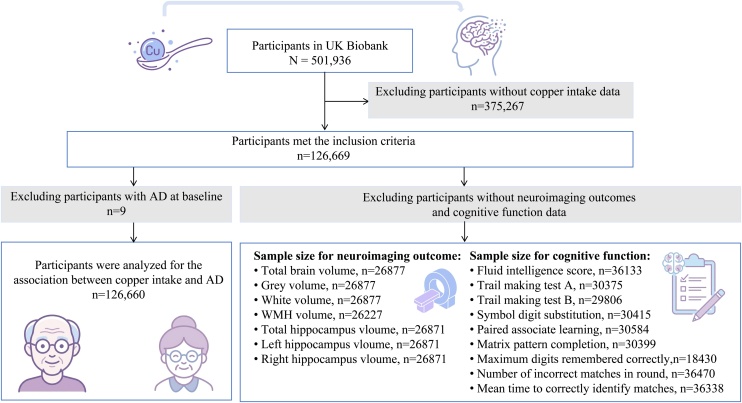
Selection of study participants in the UK Biobank.

Of all eligible participants, we first excluded those with missing data on dietary copper intake at baseline. Subsequently, we excluded individuals with a baseline diagnosis of dementia or cognitive impairment. A total of 126,660 participants were ultimately included in the final analysis. The flowchart of the study participants’ inclusion process is presented in Fig. 1.

### 2.2 Dietary copper intake assessment

Dietary intake was assessed at baseline using a 24-h recall questionnaire (Field ID: 100052) that quantified the consumption of 206 food and 32 beverage items. Participants with valid email addresses were invited to complete this questionnaire during the baseline assessment phase (April 2009 – September 2010) and in four subsequent online follow-up rounds. Dietary copper intake (Field ID: 26043) was derived as an estimated nutrient from this dietary assessment. Importantly, the estimated copper intake reflects dietary sources only, and does not include copper intake from dietary supplements.

### 2.3 Alzheimer's disease outcomes

Incident cases of AD were identified using multiple data sources within the UKB. The primary case ascertainment relied on the algorithmically defined outcomes (Field ID 42020 for date; 42021 for source). Additionally, we utilized first-occurrence data for AD (Field ID 131036 for date; 131037 for source) to determine the initial diagnosis date. Case identification was further supplemented with hospital inpatient records (ICD-10: G30*; ICD-9: 331.8), death registry data (underlying or contributory cause of death, ICD-10: G30), and self-reported diagnoses (code 1263). The detailed definition is available at Supplemental Table S1.

### 2.4 Brain structure

Brain imaging data were obtained from the UKB imaging assessment (instance 2, conducted from 2014 onwards) at the Stockport Recruitment Centre and acquired in vivo using a standardized Siemens Skyra 3T MRI scanner. Further information can be found at UKB website. Based on prior evidence linking them to cognitive decline or AD, twelve MRI-derived measures were identified as potential candidates [9]. This study focused on seven of these as structural outcome measures: total gray matter, white matter, white matter hyperintensity (WMH), whole brain volume, and the volumes of the left and right hippocampus. In addition, WMH was further classified into periventricular and deep components to examine regional specificity. For consistent interpretation, these measures were converted to z-scores (mean = 0, SD = 1). WMH measures (including total, periventricular, and deep WMH) were natural log-transformed prior to standardization to correct for skewed distributions. The specific definition of nine outcomes was listed in Supplemental Table S2.

### 2.5 Cognitive function

In accordance with UKB guidelines, cognitive tests with good reliability and validity were selected after comprehensive consideration of participant engagement and data quality from both assessment center visits and online follow-ups [10,11]. The selected tests covered multiple domains, including fluid intelligence, matrix completion, numerical memory, reaction time, total trail making (Part A + Part B), symbol digit substitution, and pairs matching. Raw scores from all tests were standardized into z-scores (mean = 0, standard deviation = 1) to facilitate analysis, with higher z-scores indicating better cognitive performance. The specific definition of nine outcomes was listed in Supplemental Table S2.

### 2.6 Covariates

The covariates included in this study were identified through the review of relevant literature [[12], [13], [14]], including sex (female or male), age (continuous), ethnic background (Asian, White, Black, Mixed or Other), Qualification (College/University degree or Other), average total household income before tax (<18000, 18000-30999, 31000-51999 or ≥52000), alcohol drinker status (current, previous, or never), smoking status (current, previous, or never), sleep duration (<6, 6−8, or >8 h), IPAQ activity group (low, medium or high), mood swings (Yes or No), overall health rating (Excellent/Good/ or Fair/Poor), body mass index (BMI) (<18.5 kg/m^2^, 28.5−25 kg/m^2^ or >25 kg/m^2^), diabetes (Yes or No), hypertension (Yes or No), family history (Yes or No), blood lipid profiles including HDL cholesterol (continuous), LDL cholesterol (continuous), triglycerides (continuous), and total cholesterol (continuous).

To further assess genetic predisposition, we included genetic risk variables as covariates in the model, including the polygenic risk score (PRS) for AD and the apolipoprotein E (APOE) genotype. All genetic data underwent quality control and imputation by the UKB. The AD PRS was constructed based on a previously described method from a UKB study [15], with higher scores indicating greater genetic susceptibility to AD. The PRS was categorized into low, medium, and high groups. We also APOE ε4 allele, which is a well-established genetic risk factor for dementia, including AD [16,17]. The two single nucleotide polymorphisms (SNPs) defining the APOE genotype (rs429358 and rs7412) were not included in the variant set used for PRS calculation. The APOE ε4 allele was incorporated as a linear variable coded as 0, 1, or 2 based on the number of alleles. Additionally, participants were dichotomized into carriers and non-carriers based on the presence or absence of the ε4 allele.

Additionally, to reduce the impact of confounding factors, in the analysis of the association between copper intake and brain structure, we further adjusted for the following confounders: head size (based on volumetric scaling from the T1 head image to the standard atlas), head motion, head position (X, Y, Z brain center of gravity, and table position), and imaging center.

### 2.7 Statistical analysis

Descriptive characteristics of the study participants were presented as mean (standard deviation), median (interquartile range), or number (percentage, %). Participants were categorized into four groups based on copper intake: Q1 (0–<1.0 mg/day), Q2 (1.0–<2.0 mg/day), Q3 (2.0–<3.0 mg/day), and Q4 (≥3.0 mg/day). Cox proportional hazards regression models were employed to examine the association between dietary copper intake and the incidence of AD, with hazard ratios (HRs) and 95% confidence intervals (CIs) calculated. Three models were constructed: Model 1 was unadjusted; Model 2 adjusted for the covariates in Model 1 plus household income, educational attainment, ethnicity, age, and sex; Model 3 further adjusted for the covariates in Model 2 plus sleep patterns, mood fluctuations, self-rated health status, family history, smoking status, alcohol consumption, BMI, IPAQ scores, cholesterol levels, glucose levels, HDL, LDL, triglyceride levels, hypertension, diabetes, PRS groups, and APOE ε4 carrier status. Furthermore, restricted cubic spline analysis was performed based on the multivariable-adjusted Cox regression, with knots set at the 5th, 35th, 65th, and 95th percentiles, to visualize the linear or nonlinear relationship between baseline copper intake levels and AD. The analysis was conducted using the “rms” package (version 6.7.1).

We conducted sensitivity analyses to evaluate the robustness and reliability of our findings. First, to mitigate potential reverse causality, we conducted sensitivity analyses by excluding participants who developed AD within the first 2 and 5 years of follow-up. Second, we performed additional adjustments for covariates: building on the original model, we further included vitamin and mineral supplements, as well as mineral and other dietary supplements. Additionally, we carried out subgroup analyses to examine potential effect modifications based on sex, age (<65 years, ≥65 years), smoking status (current or former smoker, never smoker), alcohol consumption status (current or former drinker, never drinker), BMI (<25 kg/m², ≥25 kg/m²), PRS group (low, intermediate, high), and APOE ε4 carrier status.

In addition to the primary analysis on AD incidence, we further investigated the association between copper intake and intermediate phenotypes, including brain structure and cognitive performance. In this part of the analysis, standardized z-scores derived from brain structural and cognitive function test data were used as effect values to assess the impact of copper intake. To examine potential nonlinear relationships between copper intake and these effect values, restricted cubic spline (RCS) models were applied, with knots placed at the 5th, 35th, 65th, and 95th percentiles of copper intake. If the RCS regression results indicated a nonlinear association, inflection points between copper intake and brain structure or cognitive function were further estimated. Dose–response curves were subsequently plotted to visually represent the relationship between copper intake and the standardized scores of brains structural and cognitive function tests.

All statistical analyses in this study were carried out using R (version 4.1.2) software. All analyses were bilateral with a test level *α* of 0.05, and a difference of *P* < 0.05 was considered statistically significant.

## 3 Results

### 3.1 Baseline characteristics

This analysis included 126,660 participants (56,053 males and 70,607 females). The mean [median] age at baseline was 56.14 ± 7.83 years, and the median dietary copper intake was 1.33 mg/day (IQR: 1.10–1.61). Table 1 shows the characteristics of the overall study population and across quartiles (Q1-Q4) of dietary copper intake. These characteristics are further stratified by AD in Supplementary Table S3. Significant differences (with *P* < 0.001 for most variables) were observed in most baseline characteristics across the quartiles of dietary copper intake. Participants with higher copper intake (Q3 and Q4) were more likely to be male, slightly older, and have a higher proportion of university-level education. Conversely, the proportions of females and participants in the lowest household income (<18,000) were highest in the lowest copper intake group (Q1). Significant variations were also observed in lifestyle and health-related factors. Current drinkers and individuals engaging in IPAQ were more prevalent in the higher copper intake groups (Q3 and Q4). In contrast, the proportions of current smokers and individuals reporting poor or fair overall health were highest in the Q1 group. Furthermore, levels of LDL, Cholesterol, and triglycerides exhibited a distinct pattern, with the highest levels observed in Q2 and a decreasing trend towards Q4. The prevalence of hypertension and diabetes was slightly higher in Q4 compared to Q2 and Q3. No significant difference was found in the distribution of the PRS groups (*P* = 0.116), and only modest variation was observed in APOE ε4 carrier status across the copper intake quartiles. The distribution of genetic risk factors did not differ significantly across groups.

**Table 1:** Baseline characteristics by quartile of dietary copper intake in the study of Alzheimer's disease risk from the UK Biobank cohort (*N* = 126,660).

	Overall (*N* = 126660)	Q1 (n = 20427)	Q2 (n = 95081)	Q3 (n = 10293)	Q4 (n = 859)	*P*
Sex (%)						<0.001
Female	70607 (55.75)	13698 (67.06)	52247 (54.95)	4350 (42.26)	312 (36.32)	
Male	56053 (44.25)	6729 (32.94)	42834 (45.05)	5943 (57.74)	547 (63.68)	
Age (mean (SD))	56.14 (7.83)	55.09 (7.83)	56.29 (7.81)	56.74 (7.83)	56.84 (7.95)	<0.001
Ethnic background (%)						<0.001
Asian	1519 (1.20)	392 (1.92)	975 (1.03)	142 (1.38)	10 (1.16)	
Black	1018 (0.80)	293 (1.43)	622 (0.65)	86 (0.84)	17 (1.98)	
White	122261 (96.53)	19402 (94.98)	92179 (96.95)	9867 (95.86)	813 (94.64)	
Mixed	686 (0.54)	111 (0.54)	488 (0.51)	84 (0.82)	3 (0.35)	
Other	1176 (0.93)	229 (1.12)	817 (0.86)	114 (1.11)	16 (1.86)	
Qualifications (%)						<0.001
College or University degree	20107 (15.92)	2820 (13.88)	15235 (16.07)	1892 (18.42)	160 (18.67)	
Other	106154 (84.08)	17496 (86.12)	79581 (83.93)	8380 (81.58)	697 (81.33)	
Household income (%)						<0.001
<18000	16018 (13.92)	2879 (15.75)	11552 (13.35)	1427 (15.08)	160 (20.70)	
18000∼30999	27376 (23.80)	4340 (23.74)	20492 (23.69)	2364 (24.98)	180 (23.29)	
31000∼51999	33355 (29.00)	5229 (28.60)	25161 (29.08)	2761 (29.17)	204 (26.39)	
>=52000	38286 (33.28)	5836 (31.92)	29308 (33.88)	2913 (30.78)	229 (29.62)	
Drinking status (%)						<0.001
Never	3631 (2.87)	787 (3.86)	2557 (2.69)	261 (2.54)	26 (3.03)	
Previous	3692 (2.92)	732 (3.59)	2586 (2.72)	335 (3.26)	39 (4.55)	
Current	119247 (94.21)	18890 (92.56)	89873 (94.59)	9691 (94.21)	793 (92.42)	
Smoking status (%)						<0.001
Never	72295 (57.20)	11453 (56.21)	54686 (57.63)	5693 (55.47)	463 (54.03)	
Previous	45298 (35.84)	6933 (34.03)	34150 (35.99)	3891 (37.91)	324 (37.81)	
Current	8792 (6.96)	1989 (9.76)	6053 (6.38)	680 (6.63)	70 (8.17)	
Sleeping (%)						<0.001
<6 h	5038 (3.98)	1110 (5.44)	3446 (3.63)	427 (4.15)	55 (6.41)	
6–8 h	113689 (89.79)	17835 (87.35)	85857 (90.32)	9247 (89.88)	750 (87.41)	
>8 h	7894 (6.23)	1474 (7.22)	5753 (6.05)	614 (5.97)	53 (6.18)	
IPAQ activity (%)						<0.001
Low	19414 (15.33)	3863 (18.91)	14210 (14.95)	1237 (12.02)	104 (12.11)	
Moderate	45718 (36.10)	7067 (34.60)	34768 (36.57)	3612 (35.09)	271 (31.55)	
High	41151 (32.49)	5620 (27.51)	31078 (32.69)	4071 (39.55)	382 (44.47)	
Missing data	20377 (16.09)	3877 (18.98)	15025 (15.80)	1373 (13.34)	102 (11.87)	
Mood swings (%)						<0.001
No	74740 (60.06)	11553 (57.68)	56692 (60.65)	6008 (59.49)	487 (57.63)	
Yes	49706 (39.94)	8475 (42.32)	36782 (39.35)	4091 (40.51)	358 (42.37)	
Overall health rating (%)	102801 (81.33)	15779 (77.49)	77919 (82.09)	8445 (82.27)	658 (77.05)	<0.001
Good–excellent	102801 (81.33)	15779 (77.49)	77919 (82.09)	8445 (82.27)	658 (77.05)	
Poor/fair	23603 (18.67)	4583 (22.51)	17004 (17.91)	1820 (17.73)	196 (22.95)	
BMI (%)						<0.001
<18.5	728 (0.58)	108 (0.53)	551 (0.58)	61 (0.59)	8 (0.93)	
≥18.5 to <25.0	49253 (38.97)	7153 (35.14)	37490 (39.51)	4311 (41.96)	299 (34.93)	
≥25.0	76391 (60.45)	13097 (64.33)	56843 (59.91)	5902 (57.45)	549 (64.14)	
Family history (%)						<0.001
No	108108 (86.49)	17638 (87.67)	81031 (86.32)	8689 (85.51)	750 (88.65)	
Yes	16890 (13.51)	2481 (12.33)	12841 (13.68)	1472 (14.49)	96 (11.35)	
HDL (median [IQR])	1.40 [1.17, 1.68]	1.40 [1.18, 1.67]	1.40 [1.17, 1.68]	1.39 [1.17, 1.67]	1.40 [1.17, 1.72]	0.837
LDL (median [IQR])	3.52 [2.96, 4.10]	3.55 [2.99, 4.15]	3.51 [2.96, 4.10]	3.46 [2.94, 4.03]	3.42 [2.87, 4.00]	<0.001
Triglycerides (median [IQR])	1.41 [1.00, 2.04]	1.39 [1.00, 2.01]	1.41 [1.00, 2.04]	1.43 [1.01, 2.08]	1.47 [1.04, 2.16]	<0.001
Cholesterol (median [IQR])	5.67 [4.95, 6.42]	5.72 [4.99, 6.50]	5.67 [4.95, 6.42]	5.58 [4.89, 6.32]	5.46 [4.77, 6.25]	<0.001
Hypertension (%)						0.005
No	92469 (73.01)	14830 (72.60)	69634 (73.24)	7385 (71.75)	620 (72.18)	
Yes	34191 (26.99)	5597 (27.40)	25447 (26.76)	2908 (28.25)	239 (27.82)	
Diabetes (%)						<0.001
No	118469 (93.53)	18907 (92.56)	89187 (93.80)	9589 (93.16)	786 (91.50)	
Yes	8191 (6.47)	1520 (7.44)	5894 (6.20)	704 (6.84)	73 (8.50)	
PRS group (%)						0.116
Low	41846 (33.78)	6672 (33.48)	31584 (33.95)	3309 (32.82)	281 (33.41)	
Medium	41380 (33.40)	6710 (33.67)	31053 (33.38)	3340 (33.13)	277 (32.94)	
High	40670 (32.83)	6549 (32.86)	30405 (32.68)	3433 (34.05)	283 (33.65)	
APOE e4 carrier (%)						0.003
No	89454 (72.08)	14487 (72.59)	67199 (72.09)	7142 (70.72)	626 (74.26)	
Yes	34656 (27.92)	5470 (27.41)	26012 (27.91)	2957 (29.28)	217 (25.74)	

Values are presented as Mean ± Standard Deviation, Median (Interquartile Range), or Number (Column Percentage). *P*-values were derived from independent *t*-tests for continuous variables and chi-square tests for categorical variables. Dietary copper intake was categorized into quartiles (Q) based on the distribution of the entire cohort. The ranges were as follows: Q1, 0-<1.0 mg/day; Q2, 1.0-<2.0 mg/day; Q3, 2.0-<3.0 mg/day; Q4, ≥3.0 mg/day.

### 3.2 Association between dietary copper intake and risk of AD

**Fig. 2:**
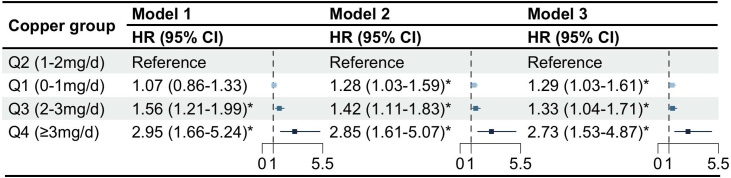
The association between dietary copper intake and Alzheimer's disease risk. The effects of dietary copper intake on Alzheimer’s disease risk. Model 1: unadjusted. Model 2: The model adjusted for Household income, Qualifications, Ethnic background, Age, Sex. Model 3: The model adjusted for Model 2 + Sleeping, Mood swings, Overall health rating, Family history, smoking status, Drinking status, BMI, IPAQ activity, Cholesterol, Glucose, HDL, LDL, Triglycerides, Hypertension, Diabetes, PRS group, APOE ε4 carrier. AD, Alzheimer’s disease; BMI, Body Mass Index; IPAQ, International Physical Activity Questionnaire; PRS, polygenic risk score; APOE, Apolipoprotein E; HR, hazard ratio; CI, confidence interval, *Significant changes (*P* < 0.05).

Based on RCS analysis, a significant non-linear U-shaped association was observed between dietary copper intake and the risk of AD, with the lowest risk identified at an intake level of 1.57 mg/day (*P* for non-linearity = 0.005; Fig. S1). Considering the U-shaped associations of copper intake with AD risk, Q2 (1–2 mg/day) was treated as the reference group when evaluating the associations between quartiles of copper intake and AD risk. After full adjustment for covariates, both lower and higher copper intake quartiles were found to be associated with an increased risk of AD compared to Q2. Specifically, individuals in Q1 (0–1 mg/day) had a 29% higher risk (HR = 1.29, 95% CI: 1.03–1.61), while those in Q3 (2–3 mg/day) showed a 33% increased risk (HR = 1.33, 95% CI: 1.04–1.71) (Fig. 2). More notably, participants in the highest intake group Q4 (≥3 mg/day) experienced a 173% increase in risk (HR = 2.73, 95% CI: 1.53–4.87). This trend across the models, with all *P* values for trend below 0.01, reveals a robust and dose-responsive association between increasing dietary copper intake and the risk of AD, even after adjusting for various confounding factors.

**Fig. 3:**
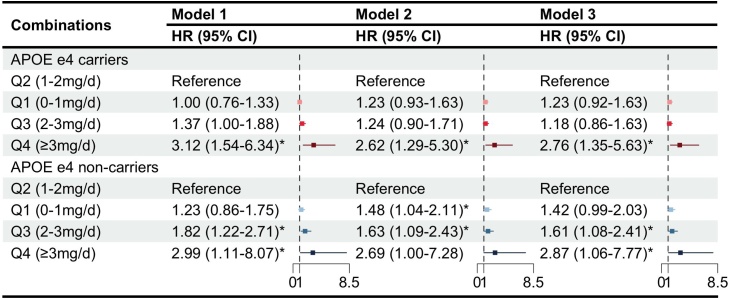
The association between dietary copper intake and Alzheimer's disease risk in APOE e4 carriers and non-carriers. (A) APOE ε4 carriers. (B) APOE ε4 non-carriers. Model 1: unadjusted. Model 2: The model adjusted for Household income, Qualifications, Ethnic background, Age, Sex. Model 3: The model adjusted for Model 2 + Sleeping, Mood swings, Overall health rating, Family history, smoking status, Drinking status, BMI, IPAQ activity, Cholesterol, Glucose, HDL, LDL, Triglycerides, Hypertension, Diabetes, PRS group, APOE e4 carrier. AD, Alzheimer’s disease; BMI, Body Mass Index; IPAQ, International Physical Activity Questionnaire; APOE, Apolipoprotein E; HR, hazard ratio; CI, confidence interval, *Significant changes (*P* < 0.05).

We conducted pre-specified stratified analyses based on age, sex, BMI, smoking status, drinking status, showed generally consistent associations across sex, age, BMI, smoking status and PRS, with no significant interactions observed, except for drinking status (*P* for interaction = 0.035) (Table. S4). Additionally, considering the influence of genetic factors on AD onset, we performed analysis for different APOE ε4 genotype carrier status (carrier, non-carrier). RCS analyses stratified by APOE ε4 status demonstrated a similar nonlinear (U-shaped) association between dietary copper intake and AD risk in both ε4 carriers and non-carriers (*P* for nonlinearity < 0.01 for both groups) (Fig. S2 & S3).When evaluating the associations by quartiles of copper intake using Q2 (1–2 mg/day) as the reference, distinct patterns emerged between carriers and non-carriers. Among APOE ε4 carriers, only those in the highest intake quartile (Q4, ≥3 mg/day) showed a significantly increased risk of AD (HR = 2.76, 95% CI: 1.35–5.63). In contrast, among non-carriers, both Q3 (2–3 mg/day; HR = 1.61, 95% CI: 1.08–2.41) and Q4 (≥3 mg/day; HR = 2.87, 95% CI: 1.06–7.77) were associated with a significantly elevated AD risk (Fig. 3). These results suggest that while high copper intake consistently elevates AD risk in both genetic subgroups, non-carriers may also exhibit increased risk at moderately high intake levels (Q3), highlighting a potential influence of APOE ε4 status on the dose-response relationship between copper intake and AD. Additional sensitivity analyses showed that excluding participants diagnosed with AD within the first 2 (Table S5) and 5 years of follow-up (Table S6) yielded results consistent with the primary analysis. Further adjustment for covariates (Table S7) also produced comparable effect estimates and preserved the overall pattern of association, supporting the robustness of the findings.

### 3.3 Association between dietary copper intake and brain structure

**Fig. 4:**
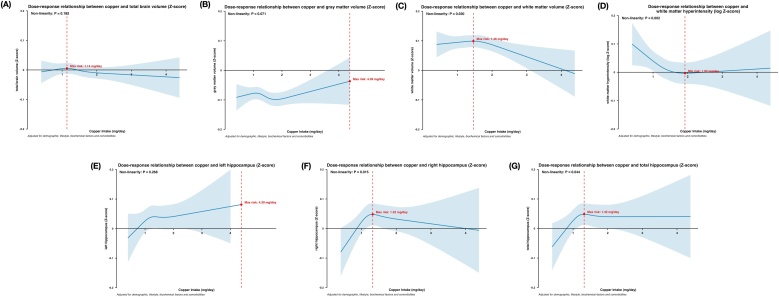
Dose-response relationship between dietary copper intake and brain structure. (A) Total brain volume. (B) Grey matter volume. (C) White matter volume. (D) White matter hyperintensity. (E) Left hippocampus. (F) Right hippocampus. (G) Total hippocampus. Restricted cubic spline for testing the hypothesis of nonlinear correlation between copper intake and brain structure. Spline curves represent Z-scores adjusted for household income, qualifications, ethnic background, age, sex, sleeping, mood swings, overall health rating, family history, smoking status, drinking status, BMI, IPAQ activity, cholesterol, glucose, HDL, LDL, triglycerides, hypertension, diabetes, PRS group, APOE ε4 carrier, head size, head position, table position and head motion. The red dashed line indicates the position where the curve inflection point occurs. AD, Alzheimer’s disease; BMI, Body Mass Index; IPAQ, International Physical Activity Questionnaire; PRS, polygenic risk score; APOE, Apolipoprotein E; CI, confidence interval.

The results of the RCS analysis revealed a significant non-liner relationship between copper intake and white matter volume, right hippocampal volume, total hippocampal volume, and white matter hyperintensities (*P* < 0.05). Fig. 4 illustrates the dose-response relationships between copper intake and these four outcomes (z-scores). Non-linear analysis showed that the change in white matter volume reached its maximum effect at a copper intake of 1.46 mg/day (Fig. 4C). Similarly, the maximum changes in right hippocampal volume and total hippocampal volume were observed at copper intake levels of 1.32 mg/day (Fig. 4F & G). In contrast, the change in white matter hyperintensities reached its minimal effect at a copper intake of 1.92 mg/day (Fig. 4D). To further examine regional specificity, periventricular and deep white matter hyperintensities were analyzed separately. Both showed significant non-linear associations with copper intake, with the highest level observed at approximately 0.4 mg/day for periventricular white matter hyperintensities and the lowest level at approximately 1.74 mg/day for deep white matter hyperintensities (Fig. S4 & S5). No significant linear or non-linear associations were found for total brain volume, gray matter volume, or left hippocampal volume (Fig. 4A, B &E).

### 3.4 Association between dietary copper intake and cognitive function

**Fig. 5:**
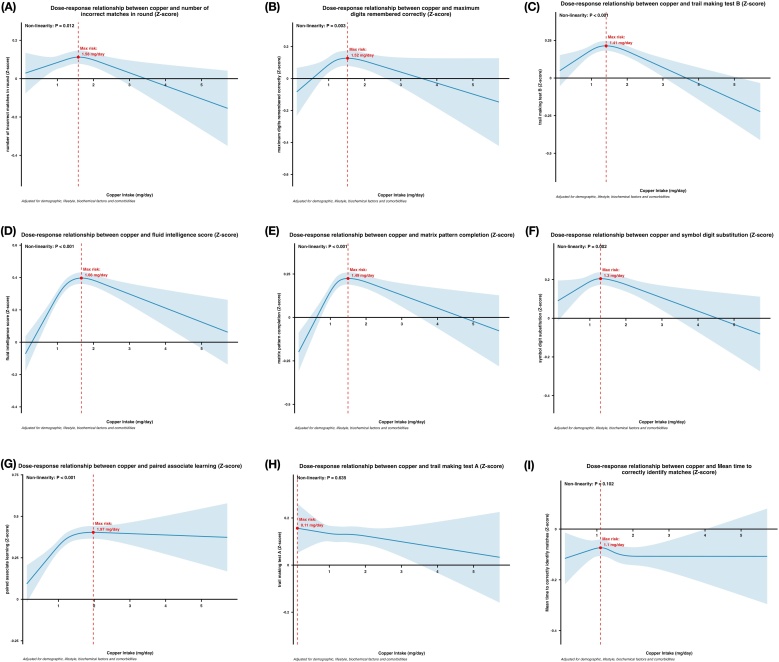
Dose-response relationship between dietary copper intake and cognition function. (A) Number of incorrect matches in round. (B) Maximum digits remember correctly. (C) Trail making test B. (D) Fluid intelligence score. (E) Matrix pattern completion. (F) Symbol digit substitution. (G) Paired associate learning. (H) Trail making test A. (I) Mean time to correctly identify matches. Restricted cubic spline for testing the hypothesis of nonlinear correlation between copper intake and brain structure. Spline curves represent Z-scores adjusted for household income, qualifications, ethnic background, age, sex, sleeping, mood swings, overall health rating, family history, smoking status, drinking status, BMI, IPAQ activity, cholesterol, glucose, HDL, LDL, triglycerides, hypertension, diabetes, PRS group, APOE ε4 carrier. The red dashed line indicates the position where the curve inflection point occurs. AD, Alzheimer’s disease; BMI, Body Mass Index; IPAQ, International Physical Activity Questionnaire; PRS, polygenic risk score; APOE, Apolipoprotein E; CI, confidence interval.

The results of the RCS analysis revealed significant non-linear associations between copper intake and the following outcomes: number of incorrect matches in round, fluid intelligence score, maximum digits remembered correctly, Trail Making Test B, Symbol Digit Substitution, Paired Associate Learning, and Matrix Pattern Completion (*P* < 0.05). Fig. 5 illustrates the dose-response relationships between copper intake and these seven outcomes (z-scores), with a general pattern of an initial increase followed by a decline. The maximum effects were observed at copper intake levels of 1.58 mg/day for number of incorrect matches (Fig. 5A), 1.52 mg/day for maximum digits remembered correctly (Fig. 5B), 1.41 mg/day for Trail Making Test B (Fig. 5C), 1.66 mg/day for fluid intelligence score (Fig. 5D), 1.49 mg/day for Matrix Pattern Completion (Fig. 5E), 1.30 mg/day for Symbol Digit Substitution (Fig. 5F) and 1.97 mg/day for Paired Associate Learning (Fig. 5G). In contrast, no significant linear or non-linear associations were found for Mean Time to Correctly Identify Matches and Trail Making Test A.

## 4 Discussion

This study is the largest to date assessing the relationship between dietary copper intake and AD in a population. The main findings of this study are summarized as follows: First, we found both excessively high and low copper intake levels were associated with increased AD risk. Second, among never and previous drinkers, the association between high dietary copper intake and AD risk was more pronounced, while no significant differences were observed in other demographic subgroups (e.g., age, sex). Third, the relationship between dietary copper intake and AD risk remained consistent across all genetic subgroups, including APOE ε4 carrier status. Finally, we found a nonlinear relationship between copper intake and both cognitive function scores and brain structure.

Our study found both low and high levels of dietary copper intake associated with an increased risk of AD. Current epidemiological evidence on the relationship between copper and AD has primarily focused on systemic copper levels, with inconsistent conclusions. A systematic review that aggregated data from 73 population-based studies found significantly higher copper levels across multiple tissue samples in AD patients compared to healthy controls (SMD = 0.37) [18], suggesting widespread dysregulation of copper metabolism in the AD population. Other studies measuring serum copper levels in AD patients have reported both significantly elevated and unchanged concentrations [19,20]. However, none of these studies evaluated the long-term dose-response relationship between dietary copper intake and AD risk. Among the limited number of dietary studies, one investigation involving 15,792 participants from four U.S. communities found that higher copper intake was associated with an increased risk of dementia, particularly in individuals with high saturated fat intake, although it did not formally assess the dose-response relationship [6]. Our study extends the existing literature by comprehensively examining dietary copper intake in relation to incident AD using intake quartiles. We observed that, compared with moderate intake (Q2, 1–2 mg/day), both lower intake (Q1, 0–1 mg/day) and higher intake levels (Q3, 2–3 mg/day; Q4, ≥3 mg/day) were associated with a significantly elevated risk of AD, with the highest risk observed in the top intake quartile. These results provide clear and clinically interpretable evidence that deviations from moderate copper intake, particularly excessive intake, may increase AD risk. Furthermore, stratified analyses indicated heterogeneity in the association between copper intake and AD risk. A significant interaction with drinking status was observed, with stronger associations among never- and former-drinkers, suggesting that lifestyle factors may modify the impact of dietary copper on AD risk. Genetic susceptibility also appeared to influence this relationship. When stratified by APOE ε4 status, high copper intake (Q4, ≥3 mg/day) was associated with increased AD risk in both carriers and non-carriers. However, non-carriers additionally exhibited a significantly elevated risk at moderately high intake levels (Q3, 2–3 mg/day), whereas ε4 carriers showed increased risk only at the highest intake level, indicating potential differences in sensitivity to dietary copper exposure. However, the optimal intake range is closely related to factors such as population differences, genetic background, and dietary patterns. Further research is still needed to validate the specific dose-response relationship between copper intake and AD risk.

From a mechanistic perspective, the association observed in this study is supported by biological evidence. Copper plays a crucial role in maintaining normal physiological functions due to its redox properties and is involved in the synthesis of copper-dependent enzymes. These include peptidyl glycine-α-amidating monooxygenase, which is involved in neuropeptide synthesis, and dopamine-β-monooxygenase, which is important for neurotransmission [21]. Copper can enter the brain through copper transporters located at the blood-brain barrier, playing a crucial role in the growth, maturation, and function of neurons [22]. On one hand, copper deficiency is associated with alterations in brain structure. It impairs the activity of copper-dependent enzymes such as cytochrome c oxidase and superoxide dismutase 1, leading to increased oxidative stress, compromised nitric oxide (NO) clearance, and accumulation of peroxynitrite, thereby damaging neuronal structural integrity [23,24]. On the other hand, excessive copper accumulation in the brain is increasingly recognized as a key pathological driver in the progression of AD. Copper overload accelerates Aβ deposition and tau hyperphosphorylation, promotes oxidative stress and neuroinflammation, and ultimately leads to neuronal dysfunction and damage [25,26]. Both Aβ and pathological tau protein have been established as critical biological markers of AD [27]. The dual pathological mechanisms of both copper deficiency and excess in brain impairment suggest a complex relationship between copper intake levels and the risk of neurodegenerative diseases.

Our study further revealed significant nonlinear associations between dietary copper intake and both brain structure and cognitive function, providing a mechanistic explanation from the perspectives of neuroimaging and cognitive phenotypes for the U-shaped relationship between dietary copper intake and AD risk. In terms of neuroimaging, we found that moderate copper intake was associated with higher white matter volume, greater hippocampal volume, and lower white matter hyperintensity burden, whereas both insufficient and excessive intake were linked to more adverse outcomes. Previous studies have also provided evidence linking copper to brain structural changes. For example, A retrospective analysis reported that higher concentrations of non–ceruloplasmin-bound copper in blood were associated with greater functional impairment, increased neuronal injury, and reduced overall brain volume [28]. Another cohort study of 2,836 children linked ambient copper exposure to increased gray matter concentration in the striatum, particularly the caudate nucleus, without overall tissue volume changes [29]. However, this study is the first to systematically reveal a nonlinear association with brain structure from the perspective of dietary intake. Of note is hippocampal volume, which, as a key region vulnerable in the early stages of AD and closely linked to memory function, provides direct neuroanatomical clues to explain the increased risk of AD associated with copper intake.

Regarding cognitive function, we similarly observed a consistent inverted U-shaped relationship. Across seven cognitive tests encompassing fluid intelligence, memory, processing speed, and executive function, cognitive performance improved with increasing copper intake up to a median level of approximately 1.3 mg/day, after which it declined. No significant association was found in two cognitive tests (the average time required to correctly identify matching items and Trail Making Test A). This suggests that the impact of copper on cognition is domain-specific, likely primarily affecting higher cognitive processes such as memory and executive function, which are closely associated with AD. Regarding cognitive function, previous studies have yielded inconsistent findings. A study by Li in 2019 reported that higher copper intake was associated with a reduced risk of poor cognitive performance, particularly in the Digit Symbol Substitution Test (DSST) and animal fluency test [5]. however, Wang's study, was the first to reveal a non-linear relationship between copper intake and cognitive function, identifying a threshold effect: when copper intake is below a certain point, it is positively correlated with cognitive performance, but when intake exceeds this point, the trend plateaus [30]. In 2025, a study by Wei using the NHANES database also found similar conclusions [31]. Our study further supports and extends this evidence by including nine cognitive outcomes that cover broader cognitive domains and by leveraging a larger sample size, thereby enhancing the robustness and generalizability of the finding.

This study systematically reveals the nonlinear association between dietary copper intake and the risk of AD, brain structure, and cognitive function from the perspective of dietary intake. This finding holds clear implications for nutritional epidemiology and public health practice. First, unlike most studies focusing on internal copper load (e.g., serum or tissue copper levels), our research directly assessed long-term dietary copper intake (a modifiable lifestyle factor), thereby providing direct evidence for AD prevention through dietary adjustments. Second, the identified U-shaped relationship and the "optimal intake range" highlight the necessity of further clarifying the appropriate dietary copper intake in nutritional guidelines, particularly for the general population and high-risk groups such as the older population and APOE ε4 carriers. Future research needs to integrate dietary patterns, genetic backgrounds, and lifestyle factors to develop personalized nutritional intervention strategies more precisely, thereby translating the "optimal intake range" into actionable preventive measures.

The primary strength of this study lies in its utilization of the UK Biobank cohort, which offers a relatively large sample size, long-term follow-up, and accurate assessment of brain-related diseases and neuroimaging features. It should be noted that this study has certain limitations. First, we identified AD cases based on hospital records and death registry data, which may have missed milder cases diagnosed in other settings. Such outcome misclassification could introduce bias toward a null association. Second, the UK Biobank is not fully representative of the general population, and the healthy volunteer selection bias may affect the magnitude and generalizability of the findings. Third, as our analysis of neuroimaging and cognitive function features relied solely on single time‑point data, we were unable to examine changes in neuroimaging and cognition over time. Fourth, dietary copper intake was assessed using 24 -h dietary recall data. To improve the reliability of exposure assessment, we restricted the analysis to participants with at least two repeated dietary measurements. Although this approach reduces random measurement error to some extent, 24-h recalls may still not fully capture long-term habitual intake of micronutrients such as copper. Therefore, some degree of within-person variability and exposure misclassification may remain, which is expected to be nondifferential and would likely attenuate the observed associations. In addition, copper intake was estimated from dietary data and therefore primarily reflects intake from food sources only, which may not fully capture total copper exposure. Finally, this study did not account for copper intake from drinking water.

## 5 Conclusions

This study reveals a U-shaped association between dietary copper intake and the risk of AD. Our findings extend previous knowledge, confirming that both high and low copper intake are risk factors for AD development in the general population. Notably, this association remained significant regardless of genetic risk. In addition, we observed significant nonlinear relationships between copper intake and certain cognitive functions as well as brain structures. These results offer dietary guidance for the prevention of AD onset and progression.

## Authors' contributions

Xintong Wu: Conceptualization, Formal analysis, Data Curation, Writing-Original Draft, Visualization. Yan Chen: Conceptualization, Methodology, Validation, Writing-Original Draft, Hao Liu: Conceptualization, Methodology, Validation, Writing-Review & Editing. Zhaochen Sun: Visualization. Ping Sun: Funding acquisition. Yuping Liu: Writing-Review & Editing. Ping Shuai: Conceptualization. Han Yang: Conceptualization. Liangliang Yang: Conceptualization. Zhengwei Wan: Conceptualization, Methodology, Writing-Original Draft, Resources, Writing-Review & Editing, Supervision, Funding acquisition.

## Consent for publication

Not applicable.

## Ethics approval and consent to participate

UK Biobank has obtained Research Tissue Bank approval from its governing Research Ethics Committee, as recommended by the National Research Ethics Service, and the study adheres to the ethical principles outlined in the Declaration of Helsinki. Written informed consent was obtained for all participants electronically.

## Declaration of Generative AI and AI-assisted technologies in the writing process

During the preparation of this work, the authors used AI-assisted tools in order to improve language and readability. After using these tools, the authors reviewed and edited the content as needed and take full responsibility for the content of the published article.

## Funding

This work was supported by the Sichuan Province Cadres Health Research Project (2026-109).

## Availability of data and materials

The data used in this study are available from the UK Biobank data resources. Permissions are required in order to gain access to the UK Biobank data resources, subject to successful registration and application process. Further information can be found on the UK Biobank website (https://www.ukbiobank.ac.uk/).

## Declaration of competing interest

There is no conflict of interest.
