# Supplementary material for: Association of dietary copper intake with Alzheimer’s disease, brain structure and cognitive function
Source: J Nutr Health Aging. 2026 May 7;30(7):100868. doi: 10.1016/j.jnha.2026.100868 (PMC13185901; doi:10.1016/j.jnha.2026.100868)
Supplement: Supplementary file 1 [file mmc1.docx]

**SUPPLEMENTARY MATERIALS**

**Association of dietary copper intake with Alzheimer's disease, brain structure, and cognitive function**

[Table S1: Definition of Alzheimer's disease from UK Biobank. 2](#_Toc227453588)

[Table S2. Field IDs used in brain structure and cognitive function analyses. 2](#_Toc227453589)

[Table S3. Characteristics of baseline information in control and case group (*N* = 126,660). 3](#_Toc227453590)

[Table S4: Subgroup Analysis of the Association between Copper Intake and Alzheimer's Disease Incidence: Cox Regression Results. 5](#_Toc227453591)

[Table S5: Sensitivity analysis for the association of copper intake with Alzheimer's Disease risk after excluding cases diagnosed within the first two years of follow-up. 7](#_Toc227453592)

[Table S6: Sensitivity analysis for the association of copper intake with Alzheimer's Disease risk after excluding cases diagnosed within the first five years of follow-up. 7](#_Toc227453593)

[Table S7: Sensitivity analysis of the association between copper intake and Alzheimer's Disease risk with additional two dietary covariates and re-imputation. 7](#_Toc227453594)

[Figure S1. Dose-response relationship between dietary copper intake and Alzheimer's Disease risk. 8](#_Toc227453595)

[Figure S2. Dose-response relationship between dietary copper intake and Alzheimer's Disease risk in APOE e4 carriers. 9](#_Toc227453596)

[Figure S3. Dose-response relationship between dietary copper intake and Alzheimer's Disease risk in APOE e4 non-carriers. 10](#_Toc227453597)

[Figure S4. Dose-response relationship between dietary copper intake and deep white matter hyperintensities. 11](#_Toc227453598)

[Figure S5. Dose-response relationship between dietary copper intake and periventricular white matter hyperintensities. 12](#_Toc227453599)

Table S1: Definition of Alzheimer's disease from UK Biobank.

| **Category** | **Alzheimer's disease** |
| --- | --- |
| **Field ID 41270: ICD-10** | G30, G301, G308, G309 |
| **Field ID 41270: ICD-10** | 3318 |
| **Category 1712: First occurrences records** | 131036: Date G30 first reported (Alzheimer’s disease) |
| **Field ID 20002: Self-reported cases** | 1263: dementia/alzheimers/cognitive impairment |
| **Field ID 40001&41002: Death register** | G30 |
| **Field ID 42020: Algorithmically defined outcomes** | - |

Hospital sources are defined by the International Classification of Diseases version ninth and tenth, respectively. Owing to limitation of sources for some diseases, we defined the date of diagnosis as the earliest one of them for each gynecological disease.

Table S2. Field IDs used in brain structure and cognitive function analyses.

| **Field ID** | **Brain structure** |
| --- | --- |
| **25010** | Volume of brain, grey+white matter |
| **25006** | Volume of grey matter |
| **25008** | Volume of white matter |
| **25019** | Volume of hippocampus (left) |
| **25020** | Volume of hippocampus (right) |
| **25781** | Total volume of white matter hyperintensities (from T1 and T2_FLAIR images) |
| **24485** | Total volume of periventricular white matter hyperintensities |
| **24486** | Total volume of deep white matter hyperintensities |
| **Field ID** | **Cognitive function** |
| **399** | number of incorrect matches in round |
| **20023** | Mean time to correctly identify matches |
| **20016** | fluid intelligence score |
| **4282** | maximum digits remembered correctly |
| **6348** | trail making test A |
| **6350** | trail making test B |
| **23324** | symbol digit substitution |
| **20197** | paired associate learning |
| **6373** | matrix pattern completion |

Table S3. Characteristics of baseline information in control and case group (*N* = 126,660).

|  | **Overall**  **（*N* = 126660）** | **No-AD**  **(n = 126041)** | **AD**  **(n = 619)** | ***P* value** |
| --- | --- | --- | --- | --- |
| Sex (%) |  |  |  | <0.001 |
| Female | 70607 (55.75) | 70307 (55.78) | 300 (48.47) |  |
| Male | 56053 (44.25) | 55734 (44.22) | 319 (51.53) |  |
| Age (mean (SD)) | 56.14 (7.83) | 56.10 (7.82) | 64.18 (4.20) | <0.001 |
| Ethnic background (%) |  |  |  | 0.234 |
| Asian | 1519 (1.20) | 1515 (1.20) | 4 (0.65) |  |
| Black | 1018 (0.80) | 1015 (0.81) | 3 (0.48) |  |
| White | 122261 (96.53) | 121653 (96.52) | 608 (98.22) |  |
| Mixed | 686 (0.54) | 684 (0.54) | 2 (0.32) |  |
| Other | 1176 (0.93) | 1174 (0.93) | 2 (0.32) |  |
| Qualifications (%) |  |  |  | 0.125 |
| College or University degree | 20107 (15.92) | 20023 (15.94) | 84 (13.59) |  |
| Other | 106154 (84.08) | 105620 (84.06) | 534 (86.41) |  |
| Household income (%) |  |  |  | <0.001 |
| <18000 | 16018 (13.92) | 15875 (13.86) | 143 (26.98) |  |
| 18000~30999 | 27376 (23.80) | 27195 (23.75) | 181 (34.15) |  |
| 31000~51999 | 33355 (29.00) | 33232 (29.02) | 123 (23.21) |  |
| >=52000 | 38286 (33.28) | 38203 (33.36) | 83 (15.66) |  |
| Drinking status (%) |  |  |  | 0.100 |
| Current | 119247 (94.21) | 118673 (94.22) | 574 (92.73) |  |
| Previous | 3692 (2.92) | 3665 (2.91) | 27 (4.36) |  |
| Drinking status (%) |  |  |  | 0.100 |
| Never | 3631 (2.87) | 3613 (2.87) | 18 (2.91) |  |
| Previous | 3692 (2.92) | 3665 (2.91) | 27 (4.36) |  |
| Current | 119247 (94.21) | 118673 (94.22) | 574 (92.73) |  |
| Smoking status (%) |  |  |  | <0.001 |
| Never | 72295 (57.20) | 71995 (57.24) | 300 (48.70) |  |
| Previous | 45298 (35.84) | 45017 (35.79) | 281 (45.62) |  |
| Current | 8792 (6.96) | 8757 (6.96) | 35 (5.68) |  |
| Sleeping (%) |  |  |  | 0.001 |
| <6h | 5038 (3.98) | 5007 (3.97) | 31 (5.01) |  |
| 6-8h | 113689 (89.79) | 113160 (89.81) | 529 (85.46) |  |
| >8h | 7894 (6.23) | 7835 (6.22) | 59 (9.53) |  |
| IPAQ activity (%) |  |  |  | <0.001 |
| high | 41151 (32.49) | 40963 (32.50) | 188 (30.37) |  |
| moderate | 45718 (36.10) | 45492 (36.09) | 226 (36.51) |  |
| low | 19414 (15.33) | 19346 (15.35) | 68 (10.99) |  |
| missing data | 20377 (16.09) | 20240 (16.06) | 137 (22.13) |  |
| Mood swings (%) |  |  |  | 0.661 |
| No | 74740 (60.06) | 74386 (60.06) | 354 (59.10) |  |
| Yes | 49706 (39.94) | 49461 (39.94) | 245 (40.90) |  |
| Overall health rating (%) |  |  |  | 0.026 |
| Good–excellent | 102801 (81.33) | 102322 (81.34) | 479 (77.76) |  |
| Poor/fair | 23603 (18.67) | 23466 (18.66) | 137 (22.24) |  |
| BMI (%) |  |  |  | 0.917 |
| <18.5 | 728 (0.58) | 724 (0.58) | 4 (0.65) |  |
| ≥18.5 to <25.0 | 49253 (38.97) | 49009 (38.97) | 244 (39.61) |  |
| ≥25.0 | 76391 (60.45) | 76023 (60.45) | 368 (59.74) |  |
| Family history (%) |  |  |  | <0.001 |
| No | 108108 (86.49) | 107685 (86.57) | 423 (69.12) |  |
| Yes | 16890 (13.51) | 16701 (13.43) | 189 (30.88) |  |
| HDL (median [IQR]) | 1.40 [1.17, 1.68] | 1.40 [1.17, 1.68] | 1.40 [1.16, 1.68] | 0.964 |
| LDL (median [IQR]) | 3.52 [2.96, 4.10] | 3.52 [2.96, 4.10] | 3.54 [2.92, 4.15] | 0.729 |
| Triglycerides (median [IQR]) | 1.41 [1.00, 2.04] | 1.41 [1.00, 2.04] | 1.47 [1.03, 2.06] | 0.166 |
| Cholesterol (median [IQR]) | 5.67 [4.95, 6.42] | 5.67 [4.95, 6.42] | 5.72 [4.92, 6.52] | 0.558 |
| Hypertension (%) |  |  |  | <0.001 |
| No | 92469 (73.01) | 92161 (73.12) | 308 (49.76) |  |
| Yes | 34191 (26.99) | 33880 (26.88) | 311 (50.24) |  |
| Diabetes (%) |  |  |  | <0.001 |
| No | 118469 (93.53) | 117941 (93.57) | 528 (85.30) |  |
| Yes | 8191 (6.47) | 8100 (6.43) | 91 (14.70) |  |
| PRS group (%) |  |  |  | <0.001 |
| Low | 41846 (33.78) | 41771 (33.88) | 75 (12.44) |  |
| Medium | 41380 (33.40) | 41237 (33.45) | 143 (23.71) |  |
| High | 40670 (32.83) | 40285 (32.67) | 385 (63.85) |  |
| APOE e4 carrier (%) |  |  |  | <0.001 |
| No | 89454 (72.08) | 89242 (72.26) | 212 (35.10) |  |
| Yes | 34656 (27.92) | 34264 (27.74) | 392 (64.90) |  |

Values are presented as Mean±Standard Deviation, Median (Interquartile Range), or Number (Column Percentage).

Table S4: Subgroup Analysis of the Association between Copper Intake and Alzheimer's Disease Incidence: Cox Regression Results.

| **Subgroup** | **Group** | **n** | **HR (95% CI)** | ***P* value** | ***P* for interaction** |
| --- | --- | --- | --- | --- | --- |
| Sex |  |  |  |  | 0.076 |
| Q1 | Female | 13698 | 1.46 (1.10–1.93) | 0.009 |  |
|  | Male | 6729 | 1.06 (0.74–1.53) | 0.747 |  |
| Q3 | Female | 4350 | 0.94 (0.60–1.48) | 0.791 |  |
|  | Male | 5943 | 1.58 (1.17–2.15) | 0.003 |  |
| Q4 | Female | 312 | 4.04 (1.78–9.20) | 0.001 |  |
|  | Male | 547 | 2.23 (0.99–5.03) | 0.053 |  |
| Age |  |  |  |  | 0.339 |
| Q1 | <65 | 17785 | 1.29 (0.94–1.76) | 0.114 |  |
|  | ≥65 | 2642 | 1.08 (0.78–1.48) | 0.640 |  |
| Q3 | <65 | 8485 | 1.68 (1.18–2.40) | 0.004 |  |
|  | ≥65 | 1808 | 1.15 (0.81–1.64) | 0.433 |  |
| Q4 | <65 | 692 | 3.29 (1.45–7.48) | 0.004 |  |
|  | ≥65 | 167 | 2.21 (0.98–4.99) | 0.057 |  |
| BMI |  |  |  |  | 0.922 |
| Q1 | < 25 | 7286 | 1.28 (0.88–1.86) | 0.199 |  |
|  | ≥ 25 | 13141 | 1.28 (0.97–1.69) | 0.080 |  |
| Q3 | < 25 | 4380 | 1.29 (0.88–1.89) | 0.186 |  |
|  | ≥ 25 | 5913 | 1.34 (0.96–1.87) | 0.083 |  |
| Q4 | < 25 | 308 | 3.37 (1.47–7.68) | 0.004 |  |
|  | ≥ 25 | 551 | 2.28 (1.01–5.15) | 0.046 |  |
| Smoking status |  |  |  |  | 0.430 |
| Q1 | Never | 11478 | 1.06 (0.74–1.50) | 0.761 |  |
|  | Previous | 6959 | 1.51 (1.11–2.06) | 0.009 |  |
|  | Current | 1990 | 1.11 (0.50–2.49) | 0.791 |  |
| Q3 | Never | 5712 | 1.30 (0.91–1.84) | 0.145 |  |
|  | Previous | 3898 | 1.51 (1.04–2.18) | 0.028 |  |
|  | Current | 683 | 0.65 (0.15–2.83) | 0.571 |  |
| Q4 | Never | 464 | 1.95 (0.72–5.26) | 0.190 |  |
|  | Previous | 325 | 3.99 (1.95–8.16) | < 0.001 |  |
|  | Current | 70 | 0.000 (0–Inf) | 0.998 |  |
| Drinking status |  |  |  |  | 0.035 |
| Q1 | Never | 788 | 3.71 (1.36–10.10) | 0.011 |  |
|  | Previous | 733 | 1.29 (0.46–3.63) | 0.631 |  |
|  | Current | 89935 | 1.22 (0.96–1.54) | 0.100 |  |
| Q3 | Never | 261 | 0.00 (0–Inf) | 0.999 |  |
|  | Previous | 337 | 2.41 (0.83–6.95) | 0.105 |  |
|  | Current | 9695 | 1.33 (1.03–1.72) | 0.031 |  |
| Q4 | Never | 26 | 21.00 (1.91–231) | 0.013 |  |
|  | Previous | 39 | 6.11 (1.29–29.00) | 0.023 |  |
|  | Current | 794 | 2.20 (1.13–4.28) | 0.020 |  |

Model3: The model adjusted for Model 2 + Sleeping, Mood swings, overall health rating, Family history, Smoking status, Drinking status, BMI, IPAQ activity, Cholesterol, Glucose, HDL, LDL, Triglycerides, Hypertension, Diabetes, PRS group, APOE e4 carrier. During each subgroup analysis, the model was adjusted accordingly, excluding the variable used as the stratification factor.

Table S5: Sensitivity analysis for the association of copper intake with Alzheimer's Disease risk after excluding cases diagnosed within the first two years of follow-up.

|  | **Total cases** | **Total sample size** | **Model 1** | ***P*** | **Model 2** | ***P*** | **Model 3** | ***P*** |
| --- | --- | --- | --- | --- | --- | --- | --- | --- |
| Q2 | 436 | 94645 | ref |  | ref |  | ref |  |
| Q1 | 98 | 20329 | 1.07 (0.86-1.33) | 0.556 | 1.28 (1.03-1.59) | 0.029 | 1.29 (1.03-1.61) | 0.024 |
| Q3 | 73 | 10220 | 1.56 (1.21-1.99) | < 0.001 | 1.42 (1.11-1.83) | 0.005 | 1.33 (1.04-1.71) | 0.024 |
| Q4 | 12 | 847 | 2.95 (1.66-5.24) | < 0.001 | 2.86 (1.61-5.07) | <0.001 | 2.73 (1.53-4.87) | < 0.001 |

Model1: unadjusted.

Model2: The model adjusted for Household income, Qualifications, Ethnic background, Age, Sex.

Model3: The model adjusted for Model 2 + Sleeping, Mood swings, overall health rating, Family history, smoking status, Drinking status, BMI, IPAQ activity, Cholesterol, Glucose, HDL, LDL, Triglycerides, Hypertension, Diabetes, PRS group, APOE e4 carrier.

Table S6: Sensitivity analysis for the association of copper intake with Alzheimer's Disease risk after excluding cases diagnosed within the first five years of follow-up.

|  | **Total cases** | **Total sample size** | **Model 1** | ***P*** | **Model 2** | ***P*** | **Model 3** | ***P*** |
| --- | --- | --- | --- | --- | --- | --- | --- | --- |
| Q2 | 427 | 94645 | ref |  | ref |  | ref |  |
| Q1 | 97 | 20329 | 1.08 (0.87-1.35) | 0.495 | 1.29 (1.03-1.61) | 0.013 | 1.30 (1.04-1.63) | 0.021 |
| Q3 | 72 | 10220 | 1.57 (1.22-2.01) | 0.001 | 1.43 (1.12-1.84) | 0.005 | 1.34 (1.05-1.73) | 0.021 |
| Q4 | 12 | 847 | 3.01 (1.69-5.35) | < 0.001 | 2.92 (1.64-5.19) | <0.001 | 2.79 (1.56-4.97) | < 0.001 |

Model1: unadjusted.

Model2: The model adjusted for Household income, Qualifications, Ethnic background, Age, Sex.

Model3: The model adjusted for Model 2 + Sleeping, Mood swings, overall health rating, Family history, smoking status, Drinking status, BMI, IPAQ activity, Cholesterol, Glucose, HDL, LDL, Triglycerides, Hypertension, Diabetes, PRS group, APOE e4 carrier.

Table S7: Sensitivity analysis of the association between copper intake and Alzheimer's Disease risk with additional two dietary covariates and re-imputation.

|  | **Total cases** | **Total sample size** | **Model 4** | ***P*** |
| --- | --- | --- | --- | --- |
| Q2 | 436 | 94645 | ref |  |
| Q1 | 98 | 20329 | 1.30 (1.04-1.63) | 0.019 |
| Q3 | 73 | 10220 | 1.33 (1.04-1.71) | 0.026 |
| Q4 | 12 | 847 | 2.72 (1.53-4.84) | < 0.001 |

Model1: unadjusted.

Model2: The model adjusted for Household income, Qualifications, Ethnic background, Age, Sex.

Model3: The model adjusted for Model 2 + Sleeping, Mood swings, overall health rating, Family history, smoking status, Drinking status, BMI, IPAQ activity, Cholesterol, Glucose, HDL, LDL, Triglycerides, Hypertension, Diabetes, PRS group, APOE e4 carrier.

Model4: The model adjusted for Model 3 + Vitamin and mineral supplements, Mineral and other dietary supplements.

Figure S1. Dose-response relationship between dietary copper intake and Alzheimer's Disease risk.


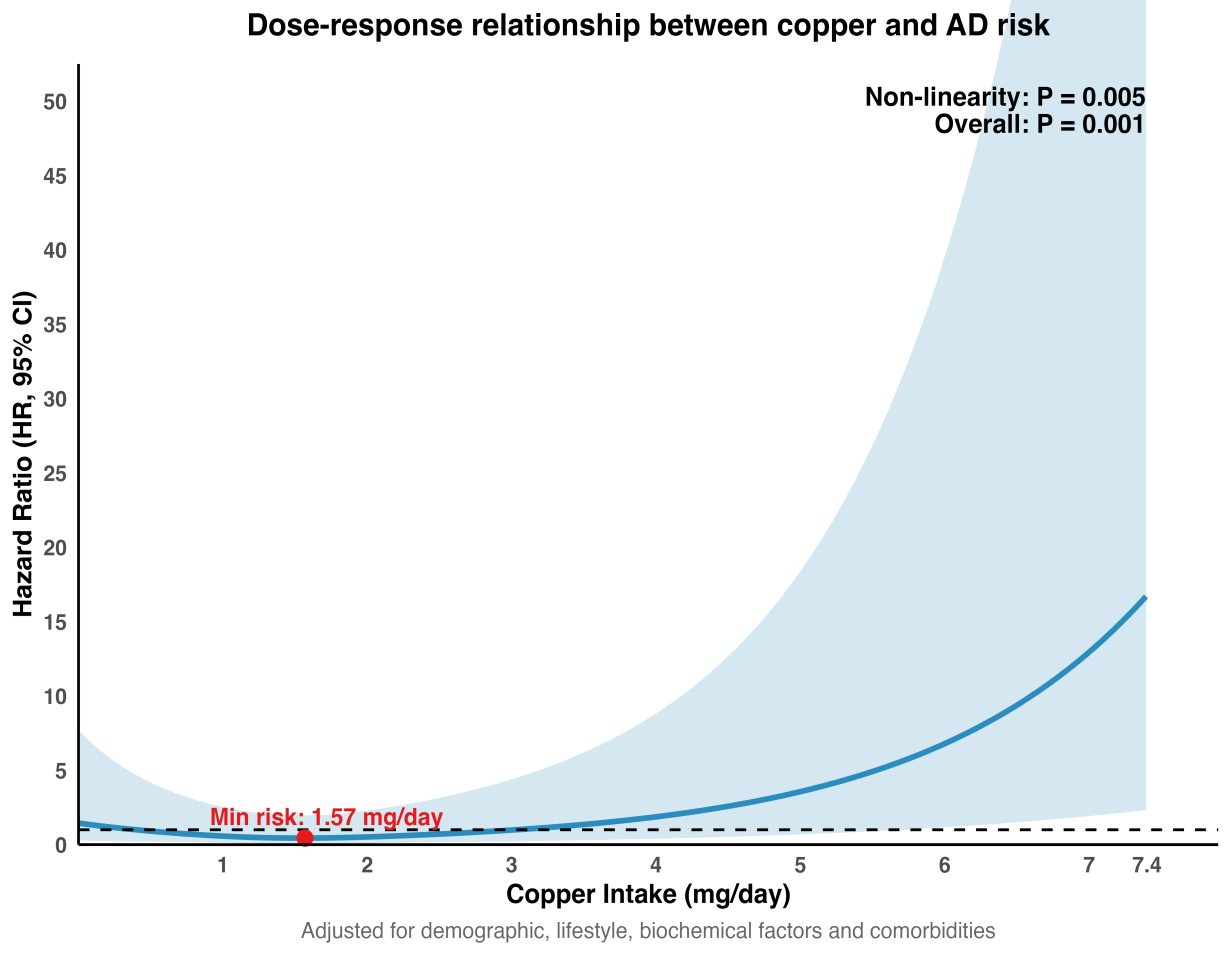


Restricted cubic splines were constructed with four knots located at the 5th, 35th, 65th, and 95th percentiles of dietary copper intake. The solid lines are fitted based on Cox-proportional hazard models adjusted for household income, qualifications, ethnic background, age, sex, sleeping, mood swings, overall health rating, family history, smoking status, drinking status, BMI, IPAQ activity, cholesterol, glucose, HDL, LDL, triglycerides, hypertension, diabetes, PRS group, APOE ε4 carrier.

AD, Alzheimer’s disease; BMI, Body Mass Index; IPAQ, International Physical Activity Questionnaire; PRS, polygenic risk score; APOE, Apolipoprotein E; HR, hazard ratio; CI, confidence interval, *Significant changes (*P* < 0.05).

Figure S2. Dose-response relationship between dietary copper intake and Alzheimer's Disease risk in APOE e4 carriers.


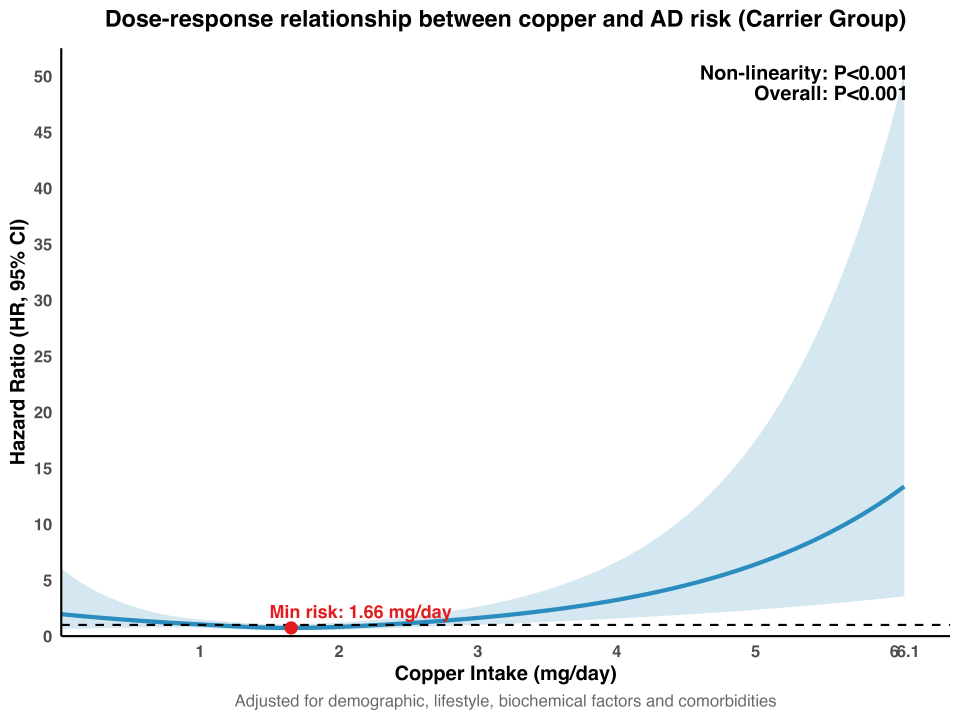


Restricted cubic splines were constructed with four knots located at the 5th, 35th, 65th, and 95th percentiles of dietary copper intake. The solid lines are fitted based on Cox-proportional hazard models adjusted for household income, qualifications, ethnic background, age, sex, sleeping, mood swings, overall health rating, family history, smoking status, drinking status, BMI, IPAQ activity, cholesterol, glucose, HDL, LDL, triglycerides, hypertension, diabetes, PRS group.

AD, Alzheimer’s disease; BMI, Body Mass Index; IPAQ, International Physical Activity Questionnaire; PRS, polygenic risk score; HR, hazard ratio; CI, confidence interval, *Significant changes (*P* < 0.05).

Figure S3. Dose-response relationship between dietary copper intake and Alzheimer's Disease risk in APOE e4 non-carriers.


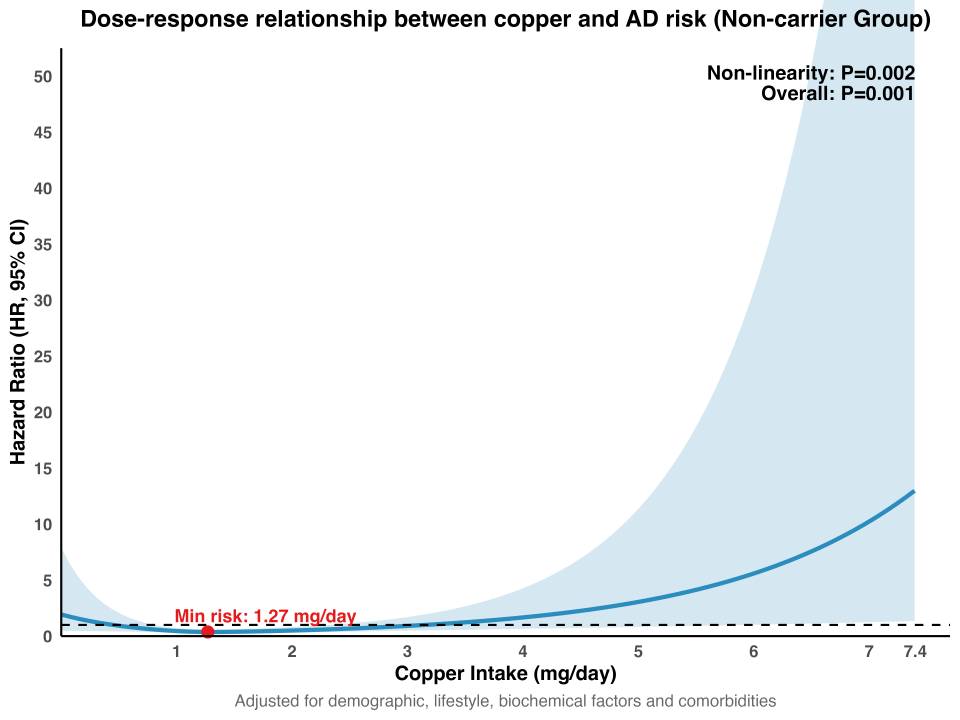


Restricted cubic splines were constructed with four knots located at the 5th, 35th, 65th, and 95th percentiles of dietary copper intake. The solid lines are fitted based on Cox-proportional hazard models adjusted for household income, qualifications, ethnic background, age, sex, sleeping, mood swings, overall health rating, family history, smoking status, drinking status, BMI, IPAQ activity, cholesterol, glucose, HDL, LDL, triglycerides, hypertension, diabetes, PRS group.

AD, Alzheimer’s disease; BMI, Body Mass Index; IPAQ, International Physical Activity Questionnaire; PRS, polygenic risk score; HR, hazard ratio; CI, confidence interval, *Significant changes (*P* < 0.05).

Figure S4. Dose-response relationship between dietary copper intake and deep white matter hyperintensities.


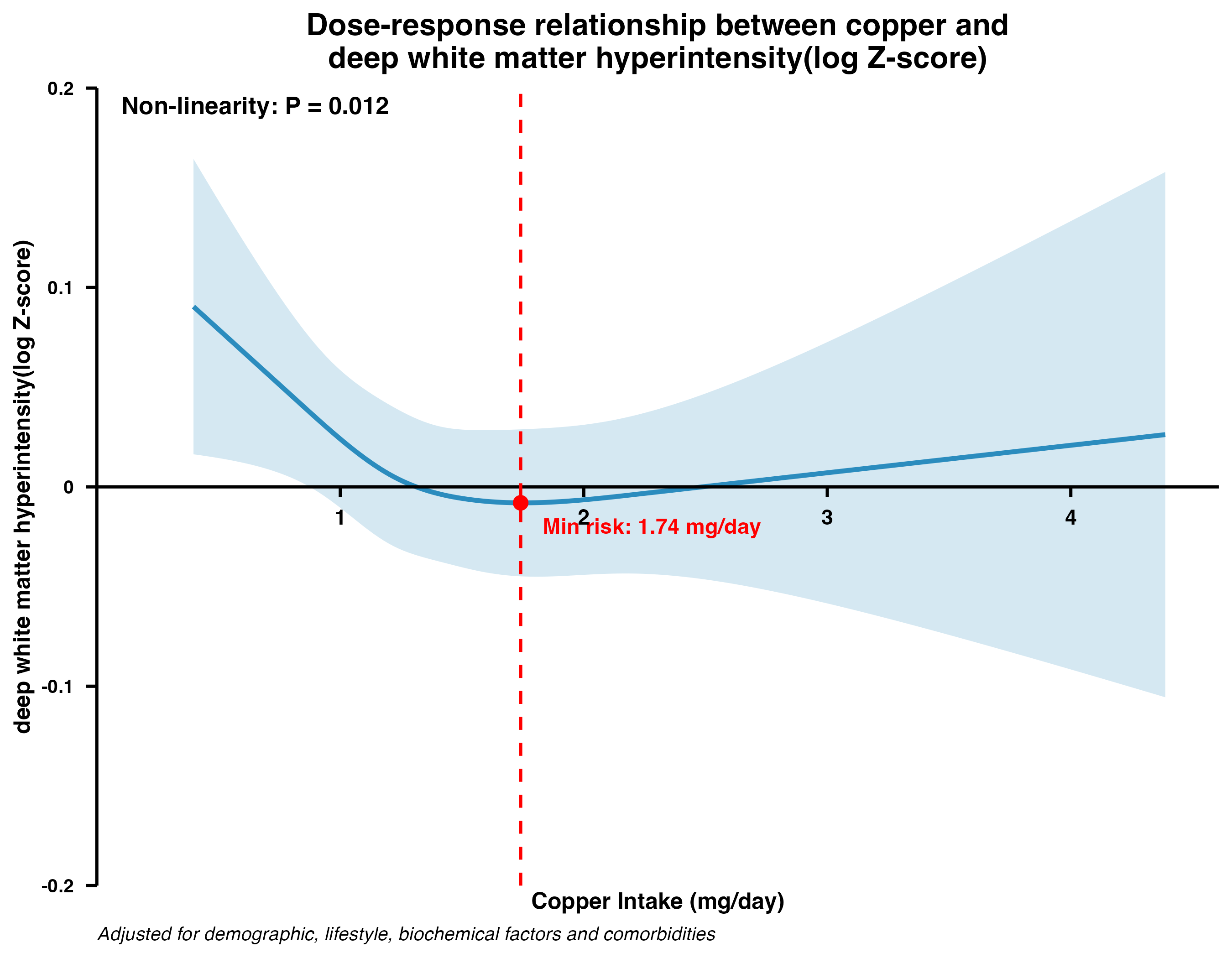


Restricted cubic spline for testing the hypothesis of nonlinear correlation between copper intake and deep white matter hyperintensities. Spline curves represent Z-scores adjusted for household income, qualifications, ethnic background, age, sex, sleeping, mood swings, overall health rating, family history, smoking status, drinking status, BMI, IPAQ activity, cholesterol, glucose, HDL, LDL, triglycerides, hypertension, diabetes, PRS group, APOE ε4 carrier, head size, head position, table position and head motion. The red dashed line indicates the position where the curve inflection points occurs.AD, Alzheimer’s disease; BMI, Body Mass Index; IPAQ, International Physical Activity Questionnaire; PRS, polygenic risk score; APOE, Apolipoprotein E; CI, confidence interval.

Figure S5. Dose-response relationship between dietary copper intake and periventricular white matter hyperintensities.


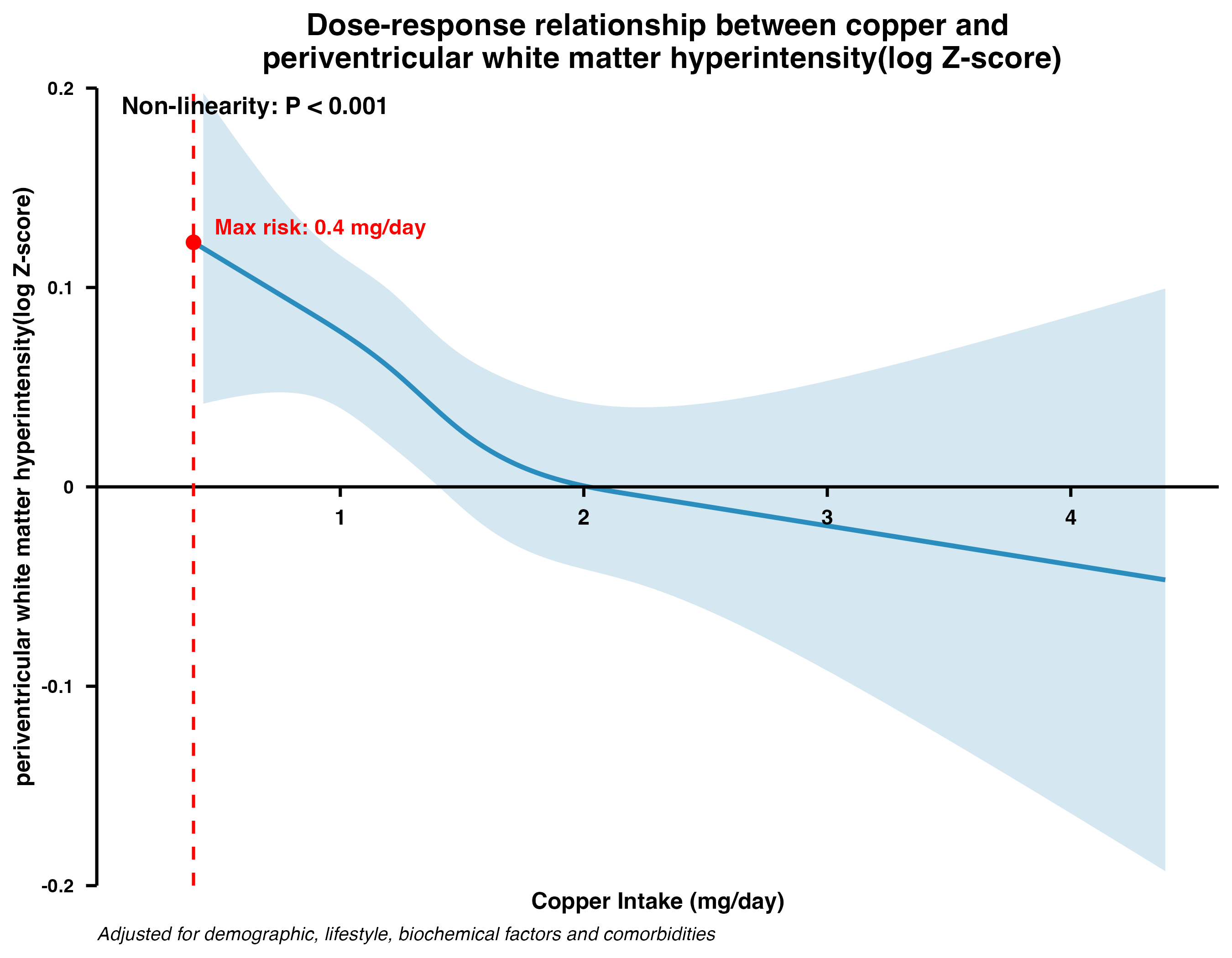


Restricted cubic spline for testing the hypothesis of nonlinear correlation between copper intake and periventricular white matter hyperintensities. Spline curves represent Z-scores adjusted for household income, qualifications, ethnic background, age, sex, sleeping, mood swings, overall health rating, family history, smoking status, drinking status, BMI, IPAQ activity, cholesterol, glucose, HDL, LDL, triglycerides, hypertension, diabetes, PRS group, APOE ε4 carrier, head size, head position, table position and head motion. The red dashed line indicates the position where the curve inflection points occurs.AD, Alzheimer’s disease; BMI, Body Mass Index; IPAQ, International Physical Activity Questionnaire; PRS, polygenic risk score; APOE, Apolipoprotein E; CI, confidence interval.
